# Evaluation of a 7-Gene Genetic Profile for Athletic Endurance Phenotype in Ironman Championship Triathletes

**DOI:** 10.1371/journal.pone.0145171

**Published:** 2015-12-30

**Authors:** Rebecca Grealy, Jasper Herruer, Carl L. E. Smith, Doug Hiller, Luke J. Haseler, Lyn R. Griffiths

**Affiliations:** 1 School of Medical Science, Griffith University, Gold Coast, Australia; 2 North Hawaii Community Hospital, John A Burns School of Medicine, University of Hawaii, Honolulu, Hawaii, United States of America; 3 Heart Foundation Research Centre, School of Physiotherapy and Exercise Science, Griffith University, Gold Coast, Australia; 4 Genomics Research Centre, Institute of Health and Biomedical Innovation, Queensland University of Technology, Kelvin Grove, Australia; University of Saarland Medical School, GERMANY

## Abstract

Polygenic profiling has been proposed for elite endurance performance, using an additive model determining the proportion of optimal alleles in endurance athletes. To investigate this model’s utility for elite triathletes, we genotyped seven polymorphisms previously associated with an endurance polygenic profile (*ACE* Ins/Del, *ACTN3* Arg577Ter, *AMPD1* Gln12Ter, *CKMM* 1170bp/985+185bp, *HFE* His63Asp, *GDF8* Lys153Arg and *PPARGC1A* Gly482Ser) in a cohort of 196 elite athletes who participated in the 2008 Kona Ironman championship triathlon. Mean performance time (PT) was not significantly different in individual marker analysis. Age, sex, and continent of origin had a significant influence on PT and were adjusted for. Only the *AMPD1* endurance-optimal Gln allele was found to be significantly associated with an improvement in PT (model p = 5.79 x 10^−17^, *AMPD1* genotype p = 0.01). Individual genotypes were combined into a total genotype score (TGS); TGS distribution ranged from 28.6 to 92.9, concordant with prior studies in endurance athletes (mean±SD: 60.75±12.95). TGS distribution was shifted toward higher TGS in the top 10% of athletes, though the mean TGS was not significantly different (p = 0.164) and not significantly associated with PT even when adjusted for age, sex, and origin. Receiver operating characteristic curve analysis determined that TGS alone could not significantly predict athlete finishing time with discriminating sensitivity and specificity for three outcomes (less than median PT, less than mean PT, or in the top 10%), though models with the age, sex, continent of origin, and either TGS or *AMPD1* genotype could. These results suggest three things: that more sophisticated genetic models may be necessary to accurately predict athlete finishing time in endurance events; that non-genetic factors such as training are hugely influential and should be included in genetic analyses to prevent confounding; and that large collaborations may be necessary to obtain sufficient sample sizes for powerful and complex analyses of endurance performance.

.

## Introduction

The ability of sport scientists to predict which athletes amongst an elite group will become world-class is limited because the interactions between biological factors, training, recovery and competitive performance are not fully understood [1]. Human physical performance depends on environmental factors such as physical training, nutrition and technological support, as well as on genetic factors such as blood lactate threshold, maximal oxygen uptake (VO_2max_), glucose/lipid metabolism, and muscular strength [2]. Over 150 DNA polymorphisms have been associated with some form of human physical performance [3]. Many of these studies have only investigated individual polymorphisms or genes; however, despite the number of genes being investigated and associated with elite endurance performance, the achievement of elite endurance performance by a relatively small number of athletes is more than likely influenced by a combination of favourable genetic alleles.

Recent studies [4–7] have proposed or utilised polygenic profiles for elite athletic performance, using a model originally outlined by Williams and Folland (2008) for optimal endurance performance [3]. While Williams and Folland’s original model contained 23 genetic polymorphisms associated with endurance performance, later models focused on smaller numbers of more strongly associated polymorphisms for endurance (seven to ten) [4, 5]. In order for comparability between models with different numbers of polymorphisms, the total genotype score (TGS) calculated generally represents the percentage of ‘optimal’ alleles for a particular phenotype. These models have been tested with other phenotypes such as success in a sporting field (in terms of the number of medals won or ranking in World and/or National Championships) [7, 8] and models with alternative polymorphisms have been proposed for speed/power performance [6, 9], mitochondrial biogenesis specific endurance models [10], and even disease/health risk models [11]. While sporting success has been previously evaluated in terms of numbers of medals won [7] or ranking in different world championship events [8], no current study has examined athlete performance within a single sporting event. However, while associations of polygenic profile polymorphisms have been well established in endurance versus power athletes, or athletes versus non-athletes, the influence of these polymorphisms on performance success within a single race event has not yet been assessed.

In this study we therefore investigate the utility of the seven-marker optimal endurance model [5] to distinguish more successful athletes (faster performance time) from less successful athletes (slower performance time) in a cohort of 196 elite endurance athletes who participated in the 2008 Kona Ironman World Championship triathlon. This cohort was initially collected in 2008 and the association of *ACTN3* Arg577Ter polymorphism analysed in this cohort in a prior study [12]. These race participants represent athletes with an extremely high level of endurance ability and present a valuable opportunity to investigate genetic endurance polymorphisms in relation to elite endurance athlete race performance. Despite the fact that participants can be classified into ‘faster’ and ‘slower’ groups based on their performance in the 2008 Kona Ironman, all qualifying athletes can be considered among the elite of worldwide endurance triathletes as the event is considered one of the most extreme endurance events in the world due to the strict qualifying requirements and the severe environmental conditions encountered during the ‘ultra’ distance race.

This study investigated whether the seven polymorphisms strongly associated with an endurance polygenic profile as described in Ruiz *et al*. 2009 [5]—*ACE* Ins/Del, *ACTN3* Arg577Ter, *AMPD1* Gln12Ter, *CKMM* 1170 bp/985+185bp, *HFE* His63Asp, *GDF8* Lys153Arg and *PPARGC1A* Gly482Ser—were individually associated with performance time (both unadjusted and adjusted for significant demographic variables) or whether the combined influence of these polymorphisms as a total genotype score (TGS) could distinguish ‘faster’ from ‘slower’ performance time of the Ironman athletes. Each of the genes included in Ruiz *et al*.’s profile is a strong candidate for involvement in endurance performance and has been found to be associated previously with improvements in physical ability. The functions of these seven genes and the impact of the profile polymorphisms on gene function are outlined below.

### *ACE* Ins/Del (rs4340)

The *ACE* 287bp Ins/Del polymorphism (I/D; rs4340) is located in intron 16 of the gene angiotensin converting enzyme (*ACE*), which is heavily involved in the cardiovascular system, in particular with blood pressure regulation. The *ACE* gene encodes a zinc metallo-carboxypeptidase that converts the inactive angiotensin I peptide into the potent vasoconstrictor angiotensin II [13, 14], which is the end product of the renin-angiotensin system (RAS) for the regulation of blood pressure. It also contributes to the regulation of blood pressure through the kinin-kallikrein system by degradation of bradykinin, a strong vasodilator [14], and is also thought to be important for muscle development due to the fact that angiotension II stimulates growth of endothelial, cardiac, and smooth muscle cells [5, 15]. The presence of the 287bp insertion (I allele) in the *ACE* gene is associated with lower levels of ACE activity in serum and tissues, with the II genotype carriers having about half the activity level of DD carriers, while ID carriers have intermediate levels [14]. The higher level of ACE activity for D allele carriers results in an increase in both angiotensin II and an increase in the metabolism of bradykinin, which, in addition to blood pressure regulation, has a significant impact on metabolic processes including uptake of glucose [15]. The D allele has also been shown to be associated with increased left ventricular hypertrophy [14] and some studies show an association with increased grip strength [9], indicating that the DD genotype may possibly be more beneficial for power sports or strength-trained athletes. Conversely, the II genotype has been found to be strongly associated with various types of endurance athletes [14, 15], and is one of the most strongly replicated associations in endurance athletes.

### *ACTN3* R577X (rs1815739)

The *ACTN3* gene encodes α-actinin-3, which is a tissue-specific actin-binding protein expressed in skeletal muscle fibers to assist in anchoring actin filaments of the sarcomere during muscle contractions. Although both α-actinin-3 and highly similar protein α-actinin-2 are both expressed in muscle, α-actinin-3 is only expressed in type II (fast-twitch, anaerobic/glycolytic) muscle fibers, which have an increased contraction speed and contraction force compared to type I (slow-twitch, oxidative) fibers [12]. The *ACTN3* Arg577Ter nonsense mutation (R577X; rs1815739) results in a truncated and non-functional protein which subsequently results in α-actinin-3 deficiency, and has been shown in knockout mouse models to decrease muscle strength and contraction force due to a decrease in the size of type II fibers. Presence of the R allele is therefore thought to improve strength and speed of contraction and has been shown to be significantly more common in sprinting athletes [9]. It has also been shown that the X allele, which results in the α-actinin-3 deficiency, shifts the type II fibers energy generation from their usual anaerobic processes to aerobic, oxidative processes, increasing the fatigue-resistance of the fibers [12]. While this suggests that the X allele may be advantageous for endurance, at a cost to speed and strength, association studies in endurance athletes have had mixed results [9]. Nevertheless, this polymorphism has a clear, replicable effect on strength and speed, and has thus been included in every profile on athletic performance.

### *AMPD1* Q12X (rs17602729)

The *AMPD1* Gln12Ter polymorphism (Q12X; rs17602729), also known as the C34T polymorphism, is located in the muscle-specific isoform of the AMP deaminase gene (*AMPD1*), which deaminates the adenosine monophosphate (AMP) that accumulates during exercise into inosine monophosphate (IMP) as part of the purine nucleotide cycle [16, 17]. An accumulation of AMP results in loss of AMP and an increase of adenosine in the tissues, which results in decreased alertness and lower time to fatigue. *AMPD1* thus assists in salvaging adenosine molecules and helping regulate the levels of IMP, AMP, adenosine diphosphate (ADP), and adenosine triphosphate (ATP) in skeletal muscles during exercise [5]. Additionally, the AMPD1 enzyme helps promote the generation of ATP from ADP by the enzyme myokinase by altering the reaction equilibrium [17], and is therefore extremely important in determining the energy availability to skeletal muscles during exercise. The substitution of a T nucleotide for a C at position 34 results in a nonsense mutation whereby a glutamine is converted to a stop codon, resulting in a truncated non-functional protein, and therefore resulting in AMPD1-deficiency. The lack of AMPD1 enzyme has been associated with an increased frequency of mild forms of myopathy post-exercise, with lower time to fatigue and muscle cramping [16], though not all individuals with AMPD1 deficiency will experience these symptoms [17]. Although the deficiency of AMPD1 was originally expected to predominantly affect short-term exercise, and although it has been associated with a lower mean anaerobic power and faster decline in power output [18], the X allele resulting in AMPD1 deficiency has been found to be about half the frequency in endurance athletes compared to controls [17]. It has since been suggested by studies examining accumulation of IMP and AMP during exercise that at the end of long endurance events when energy stores are depleted, an accumulation of AMP occurs which is necessarily converted to IMP by AMPD1 enzyme [17]. The Q allele is thus associated with an advantage for endurance performance while X allele carriers may be disadvantaged by early AMP accumulation and fatigue.

### *CKMM* 3’ UTR NcoI RFLP (rs8111989)

The gene *CKMM* contains a NcoI RFLP in the 3’ untranslated region of the gene (3’ UTR NcoI RFLP, rs8111989), resulting in two alleles named for their fragment lengths, the more common 985+185bp allele and the rarer 1170 bp allele [19], which correspond to a T to C single nucleotide substitution, respectively. The *CKMM* gene is a muscle-specific form of creatine kinase (CK) which catalyses the conversion of phospho-creatine (PCr) and ADP into creatine and ATP, as well as the reverse reaction. This CK/PCr energy buffering system acts as a temporal buffer for energy by ensuring that ATP can be quickly generated from cellular stores of ADP when required [5, 19]. It also acts as an energy ‘shuttle’ between subcellular locations. The activity of CKMM in catalysing the reaction therefore can impact on ATP availability to the muscle, which may limit performance. In fact, type I (slow twitch, oxidative) muscle fibers have been reported to show a two-fold lower CK activity compared to type II (fast-twitch, glycolytic) muscle fibers [19]. Although the NcoI RFLP is located in the 3’ UTR and thus does not result in a functional change in the CKMM protein, deletion of the *CKMM* 3’ UTR results in a change to the mRNA cellular localisation signal, which is important for correct CK/PCR shuttling [20] and which may possibly result in altered expression levels of *CKMM* due to mRNA instability [21]. Though the mechanisms by which this may affect performance are still not clear, it has been shown through performance studies that the CC genotype (1170bp/1170bp) results in a lower change in VO_2max_ (ml / kg • min) in response to endurance training, while the TT genotype results in 1.5- to 3-fold higher change in VO_2max_ [19]. This suggests that the T allele (985+185bp) may be beneficial for endurance performance [5]. The TT genotype has also been associated with an increased likelihood of extremely high blood CK levels post-exercise which may indicate damage to skeletal muscle [21] and therefore may also be involved in exercise tolerance.

### *GDF8* K153R (rs1805086)

The *GDF8* Lys153Arg polymorphism (K153R; rs1805086) is located in exon 2 of the growth differentiation factor 8 gene (*GDF8*), which is more commonly known as myostatin (abbreviation *MSTN*). Myostatin functions as a negative regulator of myoblast differentiation into muscle fibers, by signaling to increase p21, resulting in the inhibition of Cdk2 and thus the hyperphosphorylation of retinoblastoma (Rb), which then promotes cell cycle progression and thus myoblast proliferation [22, 23]. It is therefore a key factor in the determination of both the number and size of muscle fibers [22, 23], and myostatin-deficient animals, whether due to knockout, as in mouse models, or naturally deficient, as in cattle showing the ‘double-muscle’ phenotype, have been well established to exhibit up to three times as much muscle mass as wildtype [22]. Myostatin deficiency has been demonstrated to result in a similar hypertrophy of skeletal muscle in rare human cases also [24]; however, the K153R SNP, more common in humans than recessive homozygous myostatin deficiency, has also been shown to result in significant increases in skeletal muscle mass and strength for the RR genotype [23], thought to be due to alteration in binding affinity resulting in a less effective inhibition of myoblast proliferation. Its clear importance for the determination of muscle mass and strength make this marker a strong candidate for any polygenic profile of athletic performance.

### *HFE* H63D (rs1799945)

The *HFE* His63Asp polymorphism (H63D; rs1799945) is located in the hereditary haemochromatosis gene (*HFE*; standing for High Fe) which is a transmembrane protein with a key role in regulating iron absorption. The HFE protein is thought to regulate the interaction of other key molecules involved in iron uptake and circulation [25], including transferrin, a plasma protein that binds absorbed iron for circulation; the transferrin receptor (TfR, encoded by *TFRC* and *TRF2* genes), a transmembrane glycoprotein facilitating intake of transferrin-bound iron into cells; ferroportin (*FPN1* or *SLC40A1*), a transmembrane protein located on the basolateral surface of gut cells macrophages, which allows transport of absorbed iron out of cells into circulation; and hepcidin (*HAMP*), a negative regulator of iron transport that competitively binds ferroportin, preventing release of iron from cells. HFE primarily interacts with TfR by decreasing the affinity of transferrin for the TfR, thus reducing the uptake of transferring-bound iron [26, 27] as well as possibly influencing regulation of hepcidin levels, with decreases in hepcidin levels reducing the negative inhibition of ferroportin and thus increasing export for iron from gut cells into circulation and tissues [25, 28]. The H63D polymorphism has been shown to reduce the ability of the HFE protein to bind to its ligand, thereby preventing the inhibition of transferrin-TfR binding and resulting in increased transport of iron into circulation and cells [26, 27, 29]. This results in an increased level of iron, as measured by transferrin saturation (TS, or percentage of TfR bound to transferrin), serum ferritin concentration (SF, the acute-phase storage molecule for iron) [25, 29], even in the absence of additional mutations in *HFE* and the other key iron transport genes *TRF2*, *FPN1*, and *HAMP* [29]. As endurance athletes require reasonable iron levels to improve their oxygen-carrying capacity, any impairments to the iron transport mechanisms that result in a decreased level of iron, even if not at anaemic levels, may result in a poorer aerobic capacity, possibly through oxidative enzyme and respiratory protein activity [30]. Alternatively, the H63D polymorphism, by resulting in hyperferritinaemia, may have the potential to boost aerobic capacity in athletes, and indeed the D allele has been found to be at a significantly higher frequency in endurance cyclists and Olympic-class endurance runners compared to sedentary population controls [31], despite the fact that some studies have not found a significant impact on VO_2max_ from *HFE* mutations [31, 32]. The increased frequency of D allele (specifically heterozygotes) in endurance athletes therefore supports its inclusion in a polygenic model; however, due to the fact that a homozygous DD genotype may increase iron levels adversely, leading to symptoms of iron overload such as iron deposition in abdominal organs and cardiac tissue [27, 33], the heterozygous HD carrier may have the better endurance advantage, leading to its optimal weighting in Ruiz *et al*.’s polygenic profile [5].

### *PPARGC1A* G482S (rs8192678)

The *PPARGC1A* Gly482Ser polymorphism (G482S; rs8192678) is located in the peroxisome proliferator-activated receptor-γ coactivator-1α gene (*PPARGC1A*), which is a coactivator of regulatory genes for the oxidative phosphorylation (OXPHOS) pathway for generation of ATP. As endurance athletes predominantly utilise aerobic energy generation through oxidative phosphorylation, requiring higher maximal oxygen uptakes (VO_2max_) compared to sprint and power sports, the *PPARGC1A* gene could potentially impact on energy availability [34]. However, *PPARGC1A* is also involved in the activation of other pathways which may also equally be important for endurance athletes, including stimulating mitochondrial biogenesis through binding with nuclear respiratory factors NRF-1 and NRF-2 and mitochondrial transcription factors [34, 35]. PPARGC1A is also involved in glucose and lipid oxidation through its interaction with peroxisome proliferator-activated receptor α (PPARA) [34, 35]. PPARGC1A has also shown to be important for the transformation of muscle fibers to type I (slow-twitch, high levels of mitochondria) though binding with myocyte enhancer factor 2 (*MEF2*), which occurs as a result of the normal response of muscle tissue to endurance training, improving oxidative capacity and resistance to fatigue [36]. The importance of PPARGC1A is so manifold, through co-activation of differing pathways which all impact on the oxidative capacity of the skeletal muscles, that a single episode of extended endurance exercise can result in a 7- to 10-fold increase in *PPARGC1A* expression peaking within two hours [34]. The functional polymorphism G482S, which is thought to interfere with PPARGC1A binding ability, has been shown to be strongly associated with performance, with a significantly lower frequency of the S allele in endurance athletes compared to both sedentary/unfit controls [34, 35] and sprint athletes [35], highlighting the endurance advantage conferred by the more common G allele. Though there is some evidence to suggest that the S allele impede mitochondrial biogenesis by decreasing activation of mitochondrial transcription factor TFAM, stronger evidence suggests that the S allele may interfere with muscle fiber transformation as the mutation is located within the MEF2-binding site of PPARGC1A and disrupts its binding [36]. This is further supported both by mouse studies, which show that *PPARGC1A* overexpression increases type I fiber ratio while knockout models show a decrease in type I and shift to type IIx and IIb fibers, and a recent study examining human muscle biopsies, which showed a lower level of post-training type I fibers in S carriers compared to G carriers, though mitochondrial density and activity, and intracellular lipid content was not different between different genotype groups [36]. These data point to a clear advantage of G allele carriers in endurance performance and as such is an important component of any polygenic athletic profile.

## Materials and Methods

### Study population

Ethical approval was obtained from the Human Research Ethics Committee (HREC) at Griffith University (Protocol No: MSC/06/05/HREC) and Queensland University of Technology (Approval number: 1300000499) and written consent was obtained from each participant. The study population consisted of a previously described [12] cohort of 196 elite endurance triathletes, whose selection as an “elite endurance athlete” was based on participation in the 2008 Ironman World Championship triathlon. This event involves a 3.8 km swim, 180 km bike ride, and 42.2 km marathon on the Kona coast of Hawaii [37]. Questionnaires were administered at the Kona Ironman event collecting data on a variety of demographic, health, and exercise-related variables, and approximately 1–2 ml saliva was collected for each participant using saliva collection kits (OG-250 Oragene Kit, DNA Genotek Inc.). DNA was extracted from saliva samples as described previously [12] and overall finishing time (referred to henceforth as performance time, or PT) was obtained from the official Kona 2008 Ironman results [38] for 173 of the 196 recruited participants. Eligibility criteria, methodology, and cohort characteristics are described in detail elsewhere [12].

Briefly, eligibility for the Kona Ironman championship is gained by earning a qualifying place in yearly qualifying half-Ironman or full-Ironman marathons run at differing locations worldwide. Approximately three-quarters of the participants were male (N = 143, 73.0%) while about one-quarter were female (N = 53, 27.0%). Athletes originated from various countries from around the world, and were grouped according to continent of origin. Although 83.7% of athletes originated from North America (N = 104) or Europe (N = 60), although a small number did originate from Oceania (N = 23), South America (N = 6), Asia (N = 2) and Africa (N = 1). Most participants were between the ages of 30 and 50 (N = 123, 63.3%), with mean participant age 42.5 ± 11.4 yrs. Further detail on the cohort baseline characteristics and questionnaire data may be found in Grealy *et al*., 2013 [12].

### Genotyping assays

Genotyping for the seven gene polymorphisms was performed by PCR amplification followed by various assays, including agarose gel electrophoreses (AGE), restriction fragment length polymorphism (RFLP) analysis, and high resolution melt (HRM) analysis (see S1 Table for primer sequences and assay details). Briefly, the *ACE* I/D polymorphism (287 bp Alu insertion, rs4340) was genotyped by PCR amplification using a previously published primer set [39] slightly adapted. The amplicon sizes for the deletion and insertion alleles were 182bp and 470bp respectively, allowing genotype discrimination after separation by AGE. The *AMPD1* Q12X polymorphism (C>T, rs17602729) was genotyped by PCR amplification using a previously published primer set [16] followed by restriction enzyme digestion with *Hpy*CH4IV. The *GDF8* K153R polymorphism (A>G, rs1805086), the *HFE* H63D polymorphism (C>G, rs1799945), and the *PPARGC1A* G482S polymorphism (G>A, rs8192678) were all genotyped by PCR amplification using primer sets designed for this study, followed by restriction enzyme digestion with *Psp*OMI, *Bcl*I, and *Msp*I respectively. The *ACTN3* R577X polymorphism (C>T, rs1815739) had been genotyped in this cohort previously [12]; data from this study was used for this multi-gene analysis. The genotyping method in the prior study was PCR amplification followed by HRM analysis. The *CKMM Nco*I 3’-untranslated region polymorphism (A>G, rs8111989) was genotyped by PCR amplification using a HRM primer set designed for this study, followed by HRM analysis. Positive controls for each genotype were created for each assay, and were genotyped using both the original assay and an alternative assay method such as sequencing or RFLP. Both typing methods resulted in 100% concordance of genotypes, for all assays. Positive controls were subsequently included in all genotyping runs on cohort samples. Additionally, HRM assays were genotyped in duplicate, with samples re-typed in cases of disagreement between duplicates.

### Statistical analysis

Genotype frequencies were tested for conformation to Hardy-Weinberg Equilibrium (HWE), and compared to HapMap reference population frequencies using χ^2^ tests or Fisher’s exact tests where appropriate. Performance time (PT) was analysed by one-way ANOVA tests to determine whether PT differed between genotype groups for individual polymorphisms in this cohort. PTs were also used to group the athletes into two extreme phenotypes, the top 10% performers (with fastest times) and the bottom 10% performers (with slowest times). Genotype frequencies in the top and bottom 10% groups were compared using Fisher’s exact tests. The combined effect of having multiple optimal alleles was assessed using the total genotype score procedure outlined previously [5]. Briefly, each genotype for a gene is scored as 0, 1, or 2, with the most optimal genotype for endurance scored as 2. For most of the markers, the scoring system by Ruiz *et al*. assumed an additive effect of an advantageous allele, with homozygotes of the non-optimal allele assigned a score of 0 and heterozygotes with one copy of the optimal allele assigned a score of 1. The only marker that did not fit this pattern was the *HFE* H63D polymorphism, in which H/D heterozygotes were scored as 2 while the H/H homozygote was scored as 0 and the D/D homozygote was scored as 1. This was due to the prior finding that heterozygotes are significantly overrepresented in endurance athletes versus controls [5, 31]. Genotype scores for each gene are summed to a total, divided by the maximum possible score (14 for 7 genes) and divided by 100 to yield a TGS for every individual. The distribution of TGS was plotted in the overall cohort and in the 10% fastest and 10% slowest race performers, and differences in TGS were analysed in these groups by t-test analysis. PT was modeled using linear regression with stepwise forward selection, to determine whether the TGS or any of the polymorphisms individually would be a significant factor in performance time, adjusting for the demographic variables age, sex, and continent of origin (shown to significantly influence performance time in our cohort previously [12]). Due to the heterogeneity in clinical characteristics (e.g. age, sex), lifestyle characteristics (e.g. smoking status), and fitness training characteristics (e.g. estimated number of exercise hours per week), demographic, health, and exercise-related data obtained from questionnaires (described previously in Grealy *et al*., 2013) were also examined for association with PT.

Receiver operating characteristic (ROC) area under the curve (AUC) analyses were conducted to determine whether models with demographic and genetic variables could predict: (1) whether athlete performance time would be less than the median time; (2) whether athlete performance time would be less than the mean time; and (3) whether athletes would fall into the top 10% of performance times. Models included TGS only, demographic variables only, TGS and demographic variables, individual genes and demographic variables. The ROC curve is defined as a plot of test sensitivity or true positive rate (TPR) as the y coordinate versus its specificity or false positive rate (FPR) as the x coordinate. It is an effective method to evaluate the quality or the performance of an diagnostic test [40]. The clinical performance of a laboratory test can be described in terms of diagnostic accuracy, or the ability to correctly classify subjects into clinically relevant sub-groups [41]. The most common way to quantify the diagnostic accuracy of a laboratory test is to measure the area under the ROC plot or AUC. The AUC value range between 1.0 (perfect separation of the test values of the two groups) and 0.5 (no apparent distributional difference between the two groups of test values) [40, 41]. All statistical analyses were conducted using the SPSS software (IBM SPSS v. 20.0 for Windows; IBM Corporation, Somers, NY) with an α level of 0.05.

## Results

**Fig 1:**
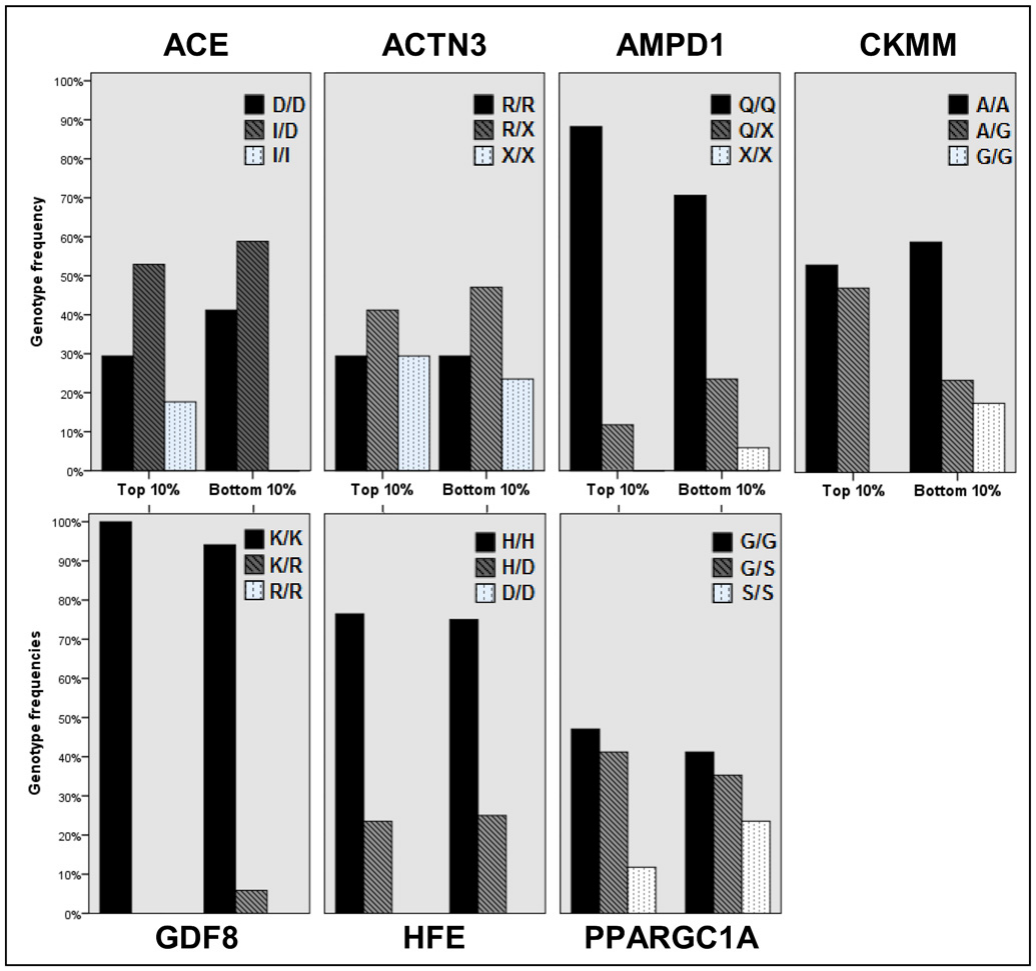
Distribution of genotypes in seven endurance related genes in the top and bottom 10% performers.

Genotyping success rate ranged from 99–100% for all markers except *HFE* (97.4% of samples successfully genotyped). The genotype distributions for all markers was found to conform with Hardy-Weinberg Equilibrium (HWE) in the overall cohort and in the subgroups of the 10% fastest and 10% slowest race performers (p > 0.05) for all groups and markers; see S2 Table. Genotype frequencies for all Ironman athletes are shown in Table 1; these concorded well with reference frequencies derived from the HapMap CEU population (Utah residents with ancestry from Northern and Western Europe) [42] and were not significantly different for any marker except *ACE* rs4340. No data was available for *ACE* rs4340 in HapMap CEU population; data shown in Table 1 is drawn from Keavney *et al*. 2000, which is a UK study involving 5934 Caucasian myocardial infarction controls [43]. The Ironman cohort had a significantly higher frequency of the D/D genotype compared to this study (Ironman 42.3% D/D compared to 27.6%; χ^2^ p = 1.68 x10^-6^). Genotype distribution was not significantly different in males and females, athletes from different continents, or athletes of different ages (see S3, S4 and S5 Tables); thus further analyses were undertaken without stratification by these groups. Genotype frequencies in the 10% fastest and 10% slowest race performers are also shown in Table 1 and Fig 1; these were not significantly different for any marker, though this is most likely due to a lack of power as n = 17 for each group. There were non-significant trends observed in genotype distribution in top and bottom performers (see S1 Fig), particularly *ACE*, with a higher frequency of the I/I genotype in the top 10% performers (17.6% compared to 0.0%); for *AMPD1*, with a higher frequency of the Q/Q genotype in the top 10% performers (88.2% compared to 70.6%); and for *CKMM*, with a lower frequency of the G/G genotype in the top 10% performers (0.0% compared to 17.6%).

**Table 1:** Genotype frequency data in the Ironman athletes and the HapMap CEU reference population [42].

				Genotype frequency, n (%)					Genotype frequency, n (%)				
Gene	rsID	Marker^a^	Genotype		HapMap CEU		All athletes	χ^2^ p		Top 10%		Bottom 10%	Exact p^c^
*ACE*	rs4340	D/I	D/D	1637^b^	(27.6%)	83	(42.3%)	1.68 ×10^−6^	5	(29.4%)	7	(41.2%)	0.278
			I/D	2980^b^	(50.2%)	92	(46.9%)		9	(52.9%)	10	(58.8%)	
			I/I	1317^b^	(22.2%)	21	(10.7%)		3	(17.6%)	0	(0.0%)	
*ACTN3*	rs1815739	R577X	R/R	22	(19.5%)	52	(26.5%)	0.29	5	(29.4%)	5	(29.4%)	1.000
			R/X	66	(58.4%)	98	(50.0%)		7	(41.2%)	8	(47.1%)	
			X/X	25	(22.1%)	46	(23.5%)		5	(29.4%)	4	(23.5%)	
*AMPD1*	rs17602729	Q12X	Q/Q	86	(76.1%)	149	(76.4%)	0.54^c^	15	(88.2%)	12	(70.6%)	0.398
			Q/X	24	(21.2%)	44	(22.6%)		2	(11.8%)	4	(23.5%)	
			X/X	3	(2.7%)	2	(1.0%)		0	(0%)	1	(5.9%)	
*CKMM*	rs8111989	3’ UTR NcoI RFLP	A/A	58	(51.3%)	93	(47.4%)	0.32	9	(52.9%)	10	(58.8%)	0.156
			A/G	49	(43.4%)	83	(42.3%)		8	(47.1%)	4	(23.5%)	
			G/G	6	(5.3%)	20	(10.2%)		0	(0.0%)	3	(17.6%)	
*GDF8*	rs1805086	K153R	K/K	58	(96.7%)	186	(95.4%)	1.00^c^	17	(100.0%)	16	(94.1%)	1.000
			K/R	2	(3.3%)	9	(4.6%)		0	(0.0%)	1	(5.9%)	
			R/R	0	(0.0%)	0	(0.0%)		0	(0.0%)	0	(0.0%)	
*HFE*	rs1799945	H63D	H/H	36	(64.3%)	138	(72.3%)	0.34^c^	13	(76.5%)	12	(75.0%)	1.000
			H/D	20	(35.7%)	51	(26.7%)		4	(23.5%)	4	(25.0%)	
			D/D	0	(0.0%)	2	(1.0%)		0	(0.0%)	0	(0.0%)	
*PPARGC1A*	rs8192678	G482S	G/G	51	(45.1%)	74	(37.9%)	0.42	8	(47.1%)	7	(41.2%)	0.811
			G/S	45	(39.8%)	84	(43.1%)		7	(41.2%)	6	(35.3%)	
			S/S	17	(15.1%)	37	(19.0%)		2	(11.8%)	4	(23.5%)	

^a^Number of successfully genotyped samples per marker: *ACE* = 196 (100%); *ACTN3* = 196 (100%); *AMPD1* = 195 (99.5%); *CKMM* = 196 (100%); *GDF8* = 195 (99.5%); *HFE* = 191 (97.4%); *PPARGC1A* = 195 (99.5%).

^b^No available data for *ACE* rs4340 in HapMap CEU population; data shown from Keavney *et al*. (2000) UK study involving 5934 Caucasian myocardial infarction controls [43].

^c^Where a small number of observations prevented use of χ^2^, Fisher’s exact test was used.

Mean performance time (PT) overall was 11 hr 44.4 min ± 1 hr 51.4 min; the fastest finishing time was 9 hr 5.3 min, while the slowest finishing time was 16 hr 55.2 min. Mean PTs and ANOVA comparisons for each genotype group are shown in Table 2. For each of the genes, the fastest PT was for: *ACE* I/I genotype (685 min); *ACTN3* R/R genotype (697 min); *AMPD1* Q/Q genotype (704 min); *CKMM* A/G (695 min); *GDF8* K/R genotype (694 min); *HFE* D/D genotype (697 min); and *PPARGC1A* G/S genotype (704 min). For *ACE* and *AMPD1*, the fastest PT corresponded with the ‘optimal’ genotype for endurance. For *CKMM*, *GDF8*, *PPARGC1A* and *HFE*, the less optimal genotype had the fastest PT. Interestingly, for *ACTN3*, the fastest PT corresponded with the genotype optimally associated with speed/power (the R/R genotype), not endurance. For *AMPD1*, a trend of increasing mean PT for decreasing number of optimal alleles was observed; however, mean PT did not significantly differ between genotype groups for any of the individual polymorphisms in this cohort (p > 0.1).

**Table 2:** Mean performance time (PT) in minutes within genotype groups.

Gene	rsID	Genotype	n	Mean PT	(SE PT)	F	p	Levene p
*ACE*	rs4340	D/D	75	704.6	(12.4)	0.655	0.521	0.304
		I/D	81	716.9	(13.2)			
		I/I	17	684.9	(23.1)			
*ACTN3*	rs1815739	R/R	45	696.7	(16.4)	0.509	0.602	0.789
		R/X	85	716.7	(12.1)			
		X/X	43	704.2	(17.2)			
*AMPD1*	rs17602729	Q/Q	132	704.4	(9.5)	1.805	0.168	0.240
		Q/X	38	716.9	(18.5)			
		X/X	2	849.4	(166.4)			
*CKMM*	rs8111989	A/A	83	717.3	(13.2)	0.954	0.387	0.144
		A/G	73	694.8	(11.2)			
		G/G	17	723.0	(31.8)			
*GDF8*	rs1805086	K/K	164	709.6	(8.8)	0.148	0.701	0.262
		K/R	8	694.0	(32.7)			
		R/R	0	-	-			
*HFE*	rs1799945	H/H	119	706.4	(10.3)	0.093	0.911	0.573
		H/D	47	714.2	(15.7)			
		D/D	2	697.2	(50.8)			
*PPARGC1A*	rs8192678	G/G	67	711.9	(14.2)	0.126	0.882	0.319
		G/S	72	703.9	(12.4)			
		S/S	33	713.6	(20.7)			

**Fig 2:**
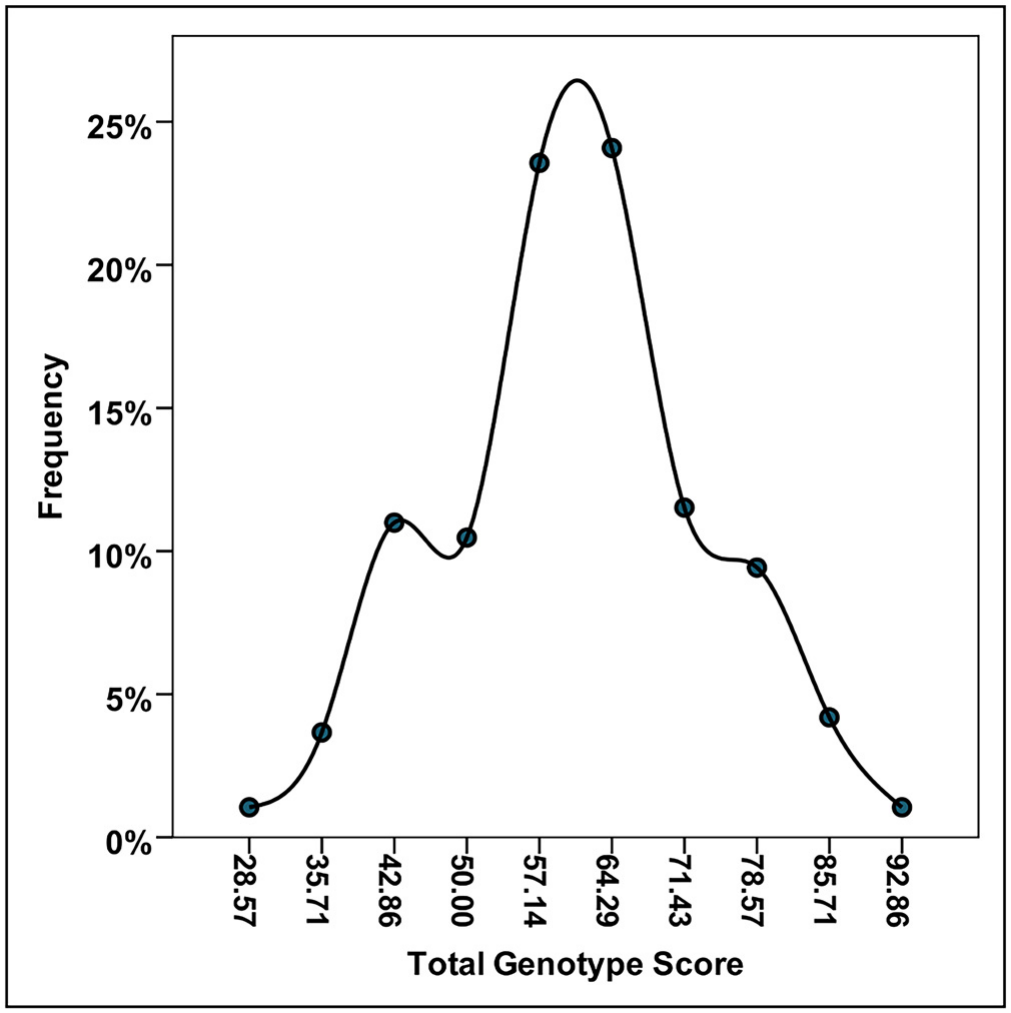
Frequency distribution of total genotype score (TGS) in overall Ironman cohort.

**Fig 3:**
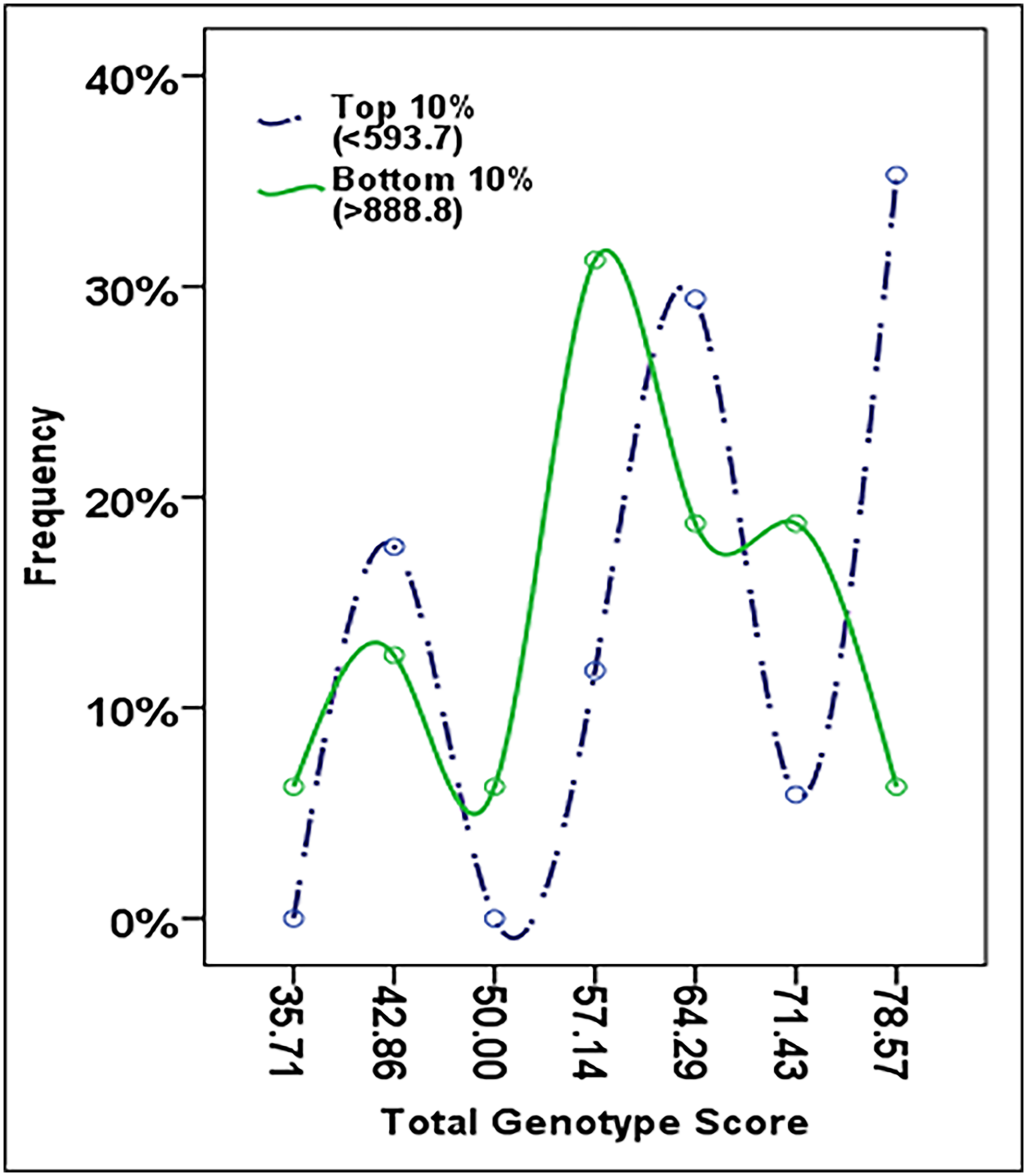
Frequency distribution of total genotype score (TGS) in top and bottom 10%.

**Fig 4:**
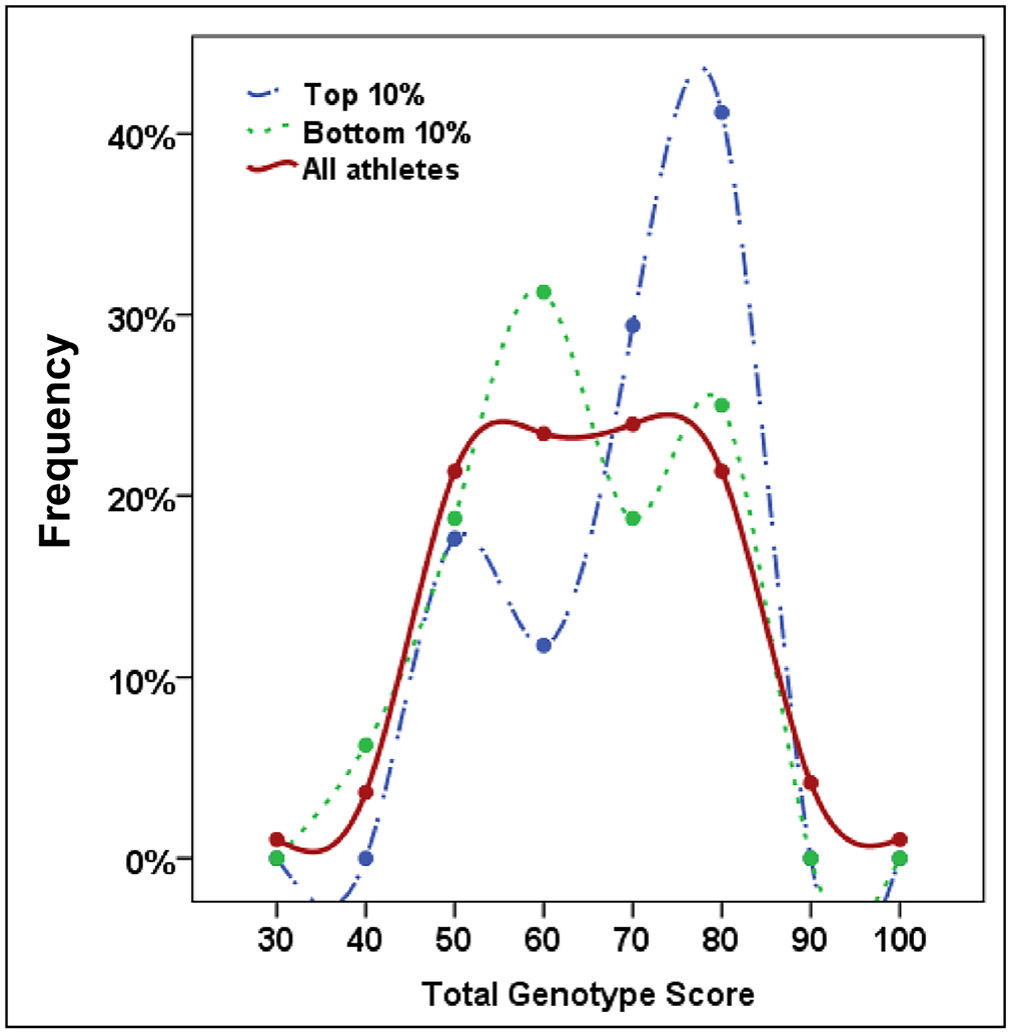
Frequency distribution of total genotype score (TGS) binned by 10-unit intervals.

Though these markers were not shown to be associated with being in the top 10% or significantly influence mean performance time individually, the combined effect of multiple optimal alleles was determined by calculating the TGS as per Ruiz *et al*. (2009), which is a percentage of optimal alleles obtained across all seven markers. In the total cohort of Ironman athletes, the mean ± SD of the TGS was 60.75 ± 12.95 (Fig 2). The TGS ranged from a minimum score of 28.6 to 92.9, with only two athletes having both the lowest and highest scores, and the distribution was both symmetrical (skewness statistic ± SE: -0.003 ± 0.18) and mesokurtic (kurtosis statistic ± SE: -0.230 ± 0.35). In the top and bottom 10% performers (Fig 3), the mean ± SD of the TGS was 65.1 ± 13.09 and 58.9 ± 11.81, respectively (n = 17 for top 10%; n = 16 for bottom 10%). The TGS distribution was also symmetrical and mesokurtic in both the top 10% (skewness statistic ± SE: -0.610 ± 0.55; kurtosis statistic ± SE: -0.734 ±1.06) and bottom 10% (skewness statistic ± SE: -0.354 ± 0.56; kurtosis statistic ± SE: -0.354 ± 1.09). The distribution in the top 10% was shifted to the right (towards higher TGS) compared to the bottom 10%. This difference was more clearly observed when TGS distribution was grouped into 10-unit intervals (Fig 4). Though mean TGS was smaller by ~6.2 units in the bottom performers compared with the top performers (or approximately one optimal allele fewer on average), this was not shown to be significant by t-test analysis (t = 1.425, df = 31, p = 0.164).

Performance time (PT) modelling using linear regression showed that clinical characteristics such as being a twin (n = 1), being a smoker (n = 1), and presence of a known disorder (n = 18) were not significantly associated with changes in PT. Occupational activity level and preferred exercise type were also shown to not significantly influence PT. There was a significant trend of decreasing mean PT with increasing estimated weekly exercise hours, with mean PT ± SD of 761 ± 126 min for athletes exercising at least 3–8 hrs per week, 701 ± 109 min for weekly exercise at least 8–12 hrs, and 682 ± 89 min for athletes exercising more than 12 hrs per week (F = 4.6, p = 0.011). However, this effect was not significant when weekly exercise hours was included in the PT regression model with other variables (β = -47.7, p = 0.224). Only the demographic variables of age (β = 4.6, p = 7.782 x 10^−12^), sex (β = 76.9, p = 2.585 x 10^−6^), and continent of origin (β = -20.4, p = 0.008) were statistically significant, accounting for most of the variance in performance time (35.1%). Regression models of individual markers followed an additive genetic model adjusted for age, sex, and continent of origin; shown in Table 3. Only the *AMPD1* marker was significantly associated with PT (model p = 5.79 x 10^−17^, *AMPD1* genotype p = 0.01). Each *AMPD1* null allele (non-optimal for endurance) resulted in an increase of about 39 minutes in PT, with X/X genotypes having an average increase of 78 min in PT compared to Q/X genotypes. The model accounted for 37.3% of the variance in PT, which was a significant improvement (F change = 6.99, p = 0.009) on the next best model of age, sex, and continent of origin alone (which accounted for 36.8% of the variance in performance). The regression model for total genotype score (Table 3) showed that TGS was not significantly associated with PT even when adjusted for age, sex, and continent of origin. The model with TGS accounted for only 34.4% of the variance in PT, which was not an improvement compared to a model with age, sex, and continent of origin alone (35.1%) or with the model of age, sex, and continent of origin with *AMPD1* genotype (37.3%).

**Table 3:** Regression models for performance time (adjusted for age, sex, continent).

Gene	N	Model R	Adjusted R^2^	Model F	Model p	Gene β	Gene p
*ACE*	173	0.603	0.348	23.97	1.07 × 10^−15^	-5.86	0.581
*ACTN3*	173	0.602	0.347	23.88	1.19 × 10^−15^	2.89	0.765
*AMPD1*	172	0.622	0.373	26.38	5.79 × 10^−17^	38.71	0.010
*CKMM*	173	0.607	0.353	24.46	5.82 × 10^−16^	-13.04	0.215
*GDF8*	172	0.605	0.351	24.12	9.24 × 10^−16^	-5.47	0.867
*HFE*	168	0.600	0.345	22.96	4.65 × 10^−15^	-13.45	0.353
*PPARGC1A*	172	0.605	0.351	24.11	9.35 × 10^−16^	0.64	0.946
TGS	168	0.600	0.344	22.86	5.22 × 10^−15^	-0.42	0.428

**Fig 5:**
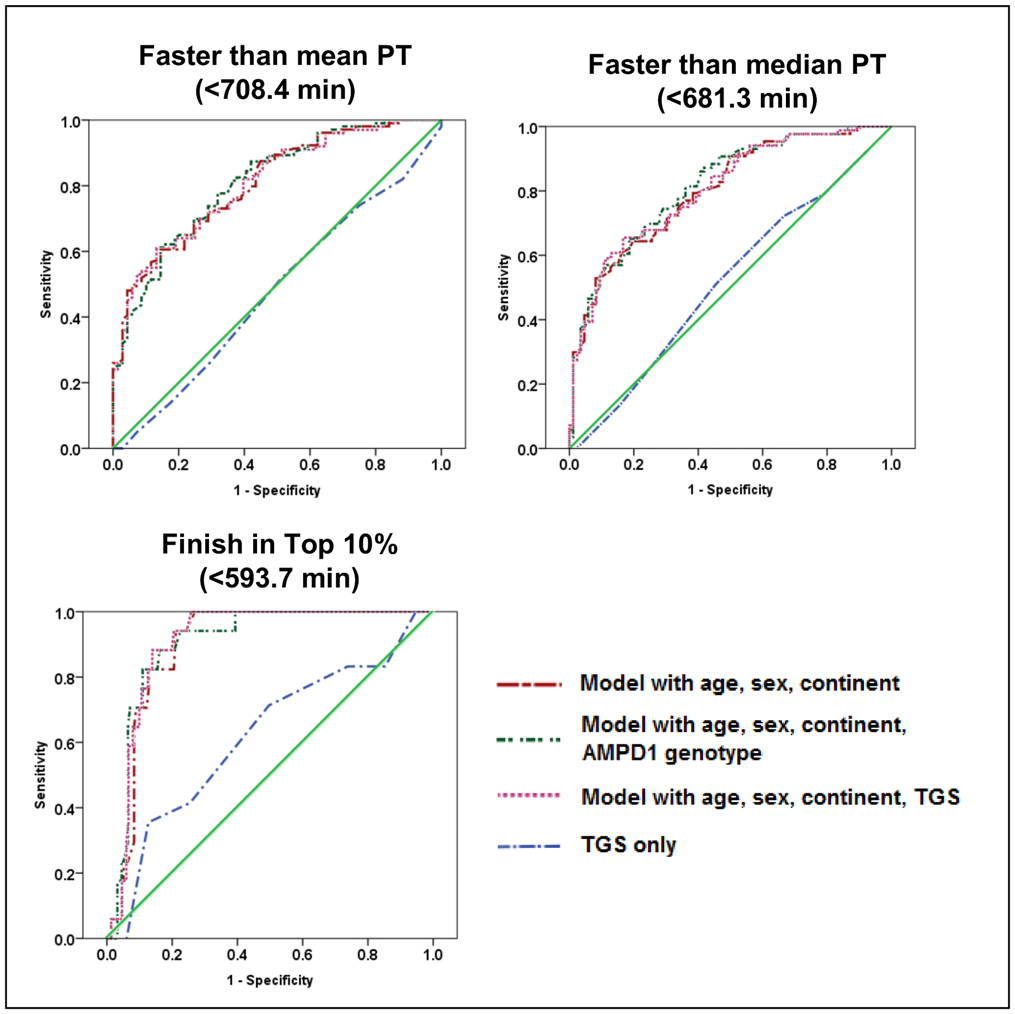
Receiver operating characteristic curves (ROC) determining potential for PT prediction using four models.

Furthermore, ROC AUC analysis determined that TGS alone could not significantly predict whether an athlete would finish in (a) less than the median PT of 681.33 min (AUC = 0.52, p = 0.674); (b) less than the mean PT of 708.39 min (AUC = 0.48, p = 0.626); or (c) the top 10% fastest PT i.e. less than 593.7 min (AUC = 0.61, p = 0.132). However, models with the demographic variables of age, sex, and continent of origin only, demographic variables and TGS, and demographic variables and *AMPD1* genotype were all found to significantly predict athlete finishing time for all three outcomes (less than median PT, less than mean PT, or in the top 10%). ROC AUC graphs for all analyses are shown in Fig 5. The model with age, sex, continent and *AMPD1* genotype was found to be the most significant for predicting whether athletes would finish in less time than both the mean and median (Median AUC = 0.82, p = 8.92 x 10^−13^, 95%CI = 0.75 to 0.88; Mean AUC = 0.81, p = 4.72 x 10^−12^, 95%CI = 0.75 to 0.87), while the model with age, sex, continent and TGS was the most significant model for predicting whether athletes would finish in the top 10% (AUC = 0.91, p = 3.50 x 10^−8^, 95%CI = 0.86 to 0.96). However, the model with age, sex, continent, and *AMPD1* genotype had similar though slightly less significant results (AUC = 0.90, p = 4.93 x 10^−8^, 95%CI = 0.85 to 0.96). Of all the ROC AUC analyses (Fig 5), the models for predicting top 10% finishers had the highest discrimination of performance in terms of sensitivity and specificity. The point where sensitivity was maximized (sensitivity = 1.000) while minimizing the false positive rate and thus maximizing specificity (specificity = 0.742) corresponded to a model value of 672.28. Using the model equation PT = (4.65 • *age*) + (79.90 • *sex*) + (-21.36 • *continent*) + (-0.42 • *TGS*) + 552.6, this would indicate that a North American male aged 35 yrs old would need a TGS of 51 or more in order to obtain the identified criteria cutoff of 672.28; however, a trade-off among the variables means that a lower TGS in combination with optimal values for the demographic variables would be equally likely to finish in the top 10%.

## Discussion

Overall, although expected genotype frequencies corresponded well with expected Caucasian frequencies from HapMap, none of the individual polymorphisms had significantly different genotype frequencies in the top and bottom 10% performers. This is perhaps due to power limitations, given that the top and bottom 10% of performers consisted of only seventeen individuals in each group for this study. However, none of the individual polymorphisms were found to significantly impact performance time when unadjusted for confounding demographic variables. Interestingly, an age-, sex- and continent of origin-adjusted analysis of *AMPD1* Gln12Ter genotype showed a significant result, with the endurance-optimal Gln allele decreasing mean performance time.

As previously reported [12], age, sex, and continent of origin were extremely significant predictors of performance time and were included in all models to control for confounding effects. This is an extremely important additional step in any genetic analysis of endurance due to the heterogeneity of athletes performing at elite levels. Some studies have avoided the main confounders of ethnicity and sex by analysing subgroups (such as males) only [5]. This approach is useful for eliminating confounders but necessarily decreases the available pool of athletes for study and may result in lack of power. Additionally, age is rarely adjusted for in endurance case-control studies, which may be an important oversight given that age was the most highly significant variable in our analyses. This is even more important when the range of age of study participants can vary (as in analyses of professional athletes). Additionally, restricting analysis by ethnic group may not remove all of the confounding present in country or continent of origin; we found a significant effect for continent of origin. This is unlikely to be due to confounding from continent-specific genetic effects as only small sample sizes were obtained from South America, Africa, and Asia, and may instead reflect continent-specific socio-economic factors relating to training availability or training type.

Indeed, training variables are an additional important factor to account for in such studies, as different training types and durations can have hugely significant impacts on athlete capabilities. In this study, fitness training characteristics were determined only through estimated weekly exercise hours (determined by exercise frequency and duration questions). However, this data alone cannot meaningfully inform the effect of athlete training on performance, as even low volume exercise may potently increase athlete endurance performance for certain training types, such as high-intensity interval training (HIT). For instance, muscle mitochondrial capacity, resting muscle glycogen, and GLUT4 protein content were all found to be improved significantly by HIT in a 2010 study, despite the fact that the training was merely six training sessions of 8–12 x 60 second intervals (with interspersed 75-second recovery periods)[44]. Furthermore, this study showed significant decreases in time to complete 50kJ and 750kJ cycling time trials with significant increases in mean power output also[44]. The benefits of HIT have even been observed for sedentary and middle-aged individuals, which obtains the health advantages of traditional endurance training with only a small time commitment[45]. Thus, explicit recording of training type, as well as training volume, are vitally important for future analyses of endurance performance.

These findings highlight the importance of including potentially confounding environmental factors in genetic analyses of athletic performance. This should not be surprising, given that while endurance endophenotypes have been shown to have high heritabilities (h^2^ = 40–60%) and while athletic status itself has also been reported to be highly heritable (h^2^ >50%) [4], non-genetic environmental factors must still contribute *at least* half of the variance in endurance phenotype. This can be due to both shared environment (such as the training provided to national-level athletes for a specific country) and non-shared environment (individual efforts in training sessions, frequency and duration of training sessions, etc.). As genetic analyses show that each allele must contribute relatively small amounts of variance to the overall phenotype compared with environmental factors [46], these types of variables should be consistently accounted for in order to prevent masking of significant genetic effects, such as we observed for *AMPD1* Gln12Ter.

Another method of preventing polymorphisms with individual small effect from escaping statistical detection is to analyse their joint effects using the TGS system. This has been used to successfully show a significant difference in genetic profile ‘favourability’ between endurance athletes versus non-athlete controls for the seven-gene endurance profile [5] or a ten-gene endurance profile [4], endurance athletes and non-athlete controls versus power athletes for a six-gene power profile [6], and endurance athletes versus power athletes and non-athlete control for a six-gene mitochondrial biogenesis endurance profile [10]. However, although the TGS distribution for our Ironman athletes (mean 60.75 ± std. dev. 12.95) was comparable to the distribution of TGS of Spanish non-athletic controls described in Ruiz *et al*. 2009 (mean 62.43 ± std. dev. 11.45), the TGS distribution in the Ironman athletes was overall lower than for Spanish endurance athletes (mean 70.22 ± std. dev. 15.58). Similar to the reported results in Spanish endurance athletes by Ruiz *et al*. 2009, we observed multiple ‘peaks’ in the distribution of the endurance athletes. The first peak was observed at a TGS ~43 and was common to both top and bottom performers; the second peak was observed at a TGS of ~57 for the bottom 10% but ~64 for the top 10%; a possible third peak was observed for top 10% performers at TGS of ~79. The difference in frequency of higher TGS for top performers compared with lower TGS for bottom performers was more clearly observed when TGS distribution was grouped into 10-unit intervals. This might suggest that there groupings of optimal alleles, perhaps, the likelihood of an optimal allele for one marker increases the likelihood of having other optimal alleles (and vice versa). Thus far, this possibility has not been explored in relation to the TGS, as what all the currently existing TGS models have in common is that they represent the proportion or percentage of ‘optimal’ alleles for a particular phenotype, and assumes an additive genetic model of allele favourability for all polymorphism except *HFE* (where the heterozygote is considered ‘most optimal’). Furthermore, the TGS follows a simple additive model of athletic advantage between different polymorphisms, which may not be the case if gene-gene and gene-environment interactions result in non-additive advantages for certain allele combinations. Several papers have already reported gene-gene interactions for small combinations of genes [4, 47, 48]; of particular interest is that performance time of South African Ironman triathletes was significantly influenced by the interaction of the *NOS3* and *BDKRB2* genes (individuals with the *NOS3* GG genotype + *BDKRB2* 19 allele were significantly slower than other combinations) [48]. More sophisticated TGS models taking such interactions into account may be necessary to accurately model genetic advantages for performance; however it is also clear that currently information on gene-gene interactions and gene-environment interactions for these genes are lacking [46]. It is also important to realise that any TGS model which accounts for gene-gene or gene-environment will become additionally complex. The power to perform such analyses may also be lacking, given that sample size has typically been an issue for elite performance studies [46, 49].

These reasons may also partly explain why TGS was not significantly associated with PT in our cohort even when adjusted for age, sex, and origin and that ROC AUC analysis determined that TGS alone could not significantly predict whether an athlete would finish in less than the median or mean or the top 10% fastest PT. Alternatively, the TGS profile for ‘optimal endurance’ may not be an appropriate profile for examining event performance as an outcome, even an endurance event. Additionally, even differing types of endurance events may show different levels of association with ‘endurance’ genes; while acknowledged as one of the most gruelling endurance events in the world, the Ironman championships require a blend of cycling, running, and swimming skills, which makes them more of a complex phenotype than single-sport endurance events such as running. Triathlons may thus require different set of ‘optimal alleles’, emphasising not only endurance-associated genes but perhaps power-associated as well. “Success” in any kind of endurance event relies, in addition to endurance capabilities, on speed and strength to outperform competitors.

Thus, in the TGS profile we employed, the *ACTN3* Arg577Ter null allele (X) was coded as the ‘optimal’ endurance allele and the X/X genotype was given a genotype score of 2, the R/X genotype given a score of 1, and the R/R genotype given a score of 0. However, the R allele is highly associated with speed and power [6], and the presence of an R allele may give an endurance event competitor an edge over an athlete with homozygous X/X genotype. In fact, Ruiz *et al*.’s 2010 speed/power profile showed three common polymorphisms to the endurance profile (*ACE* Ins/Del, *ACTN3* Arg577Ter, and *GDF8* Lys153Arg), albeit with inverse allele coding [6]. Thus, 3 out of the 14 polymorphisms used in our TGS calculation may in fact be more suitable with the power allele coded as the ‘optimal’ allele. An alternative profile for performance time may need to be investigated in order to determine a model that will predict athlete finishing time with discriminating sensitivity and specificity. Such as model may be useful in assisting with athletic training as well as helping athletes understand what factors underlie their performance, by allowing athletes to pinpoint factors to work on in order to improve performance time, as well as personalize their training to their optimal genetic profile. Before this can be done, however, more sophisticated genetic models should be investigated to ensure that the additive model is not masking gene-gene or gene-environment interactions; non-genetic factors such as training methods and duration should be recorded and included in future genetic analyses to prevent confounding; and large collaborations should be undertaken to obtain sufficient sample sizes for powerful and complex analyses of endurance performance.

## Supporting Information

S1 Fig 95% Confidence interval of mean performance time by individual marker. (TIF)

S1 Table Primer and assay information. (DOC)

S2 Table χ2 testing for conformation to Hardy-Weinberg Equilibrium (HWE). (DOC)

S3 Table χ2 testing of genotype frequencies within age, sex, and ethnicity groups. (DOC)

S4 Table Genotype distribution within male and female cohort athletes. (DOC)

S5 Table Age distribution within genotype groups. (DOC)

## Abbreviations

ACE: angiotensin-converting enzyme
ACTN3: alpha-actinin-3
AGE: agarose gel electrophoresis
AMPD1: adenosine monophosphate deaminase 1
AUC: area under the curve
BDKRB2: bradykinin receptor B2
CKMM: creatine kinase-MM
FPR: false positive rate
GDF8: growth differentiation factor 8 (also known as MSTN or myostatin)
GLUT4: glucose transporter type 4
HFE: high iron Fe, more commonly known as Human hemochromatosis gene
HIT: high-intensity interval training
HREC: Human Research Ethics Committee
HRM: high resolution melt
HWE: Hardy-Weinberg Equilibrium
NOS3: nitric oxide synthase 3
PPARGC1A: peroxisome proliferator-activated receptor gamma, coactivator 1 alpha
RFLP: restriction fragment length polymorphism
ROC: receiver operating characteristic
TGS: total genotype score
TPR: true positive rate
