# Supplementary figures and images for: Evaluation of a 7-Gene Genetic Profile for Athletic Endurance Phenotype in Ironman Championship Triathletes
Source: PLoS One. 2015 Dec 30;10(12):e0145171. doi: 10.1371/journal.pone.0145171 (PMC4696732; doi:10.1371/journal.pone.0145171)

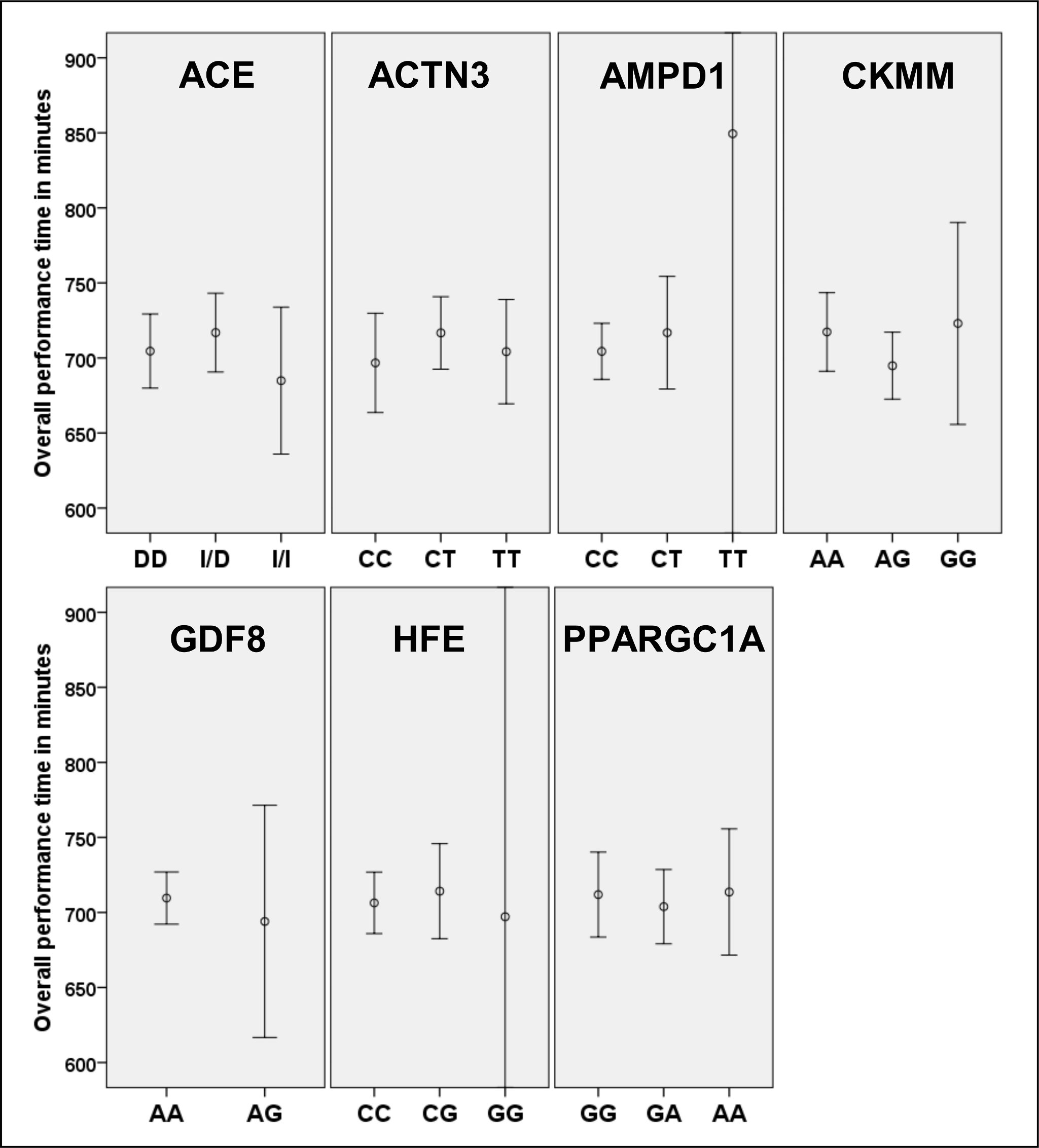

Supplement: S1 Fig — (TIF) [file pone.0145171.s001.tif]
