# Supplementary material for: Evaluation of a 7-Gene Genetic Profile for Athletic Endurance Phenotype in Ironman Championship Triathletes
Source: PLoS One. 2015 Dec 30;10(12):e0145171. doi: 10.1371/journal.pone.0145171 (PMC4696732; doi:10.1371/journal.pone.0145171)
Supplement: S1 Table — (DOC) [file pone.0145171.s002.doc]

**S1 Table: Primer and assay information**

| **Gene** | **Marker** | **rsID** | **Alleles** | **Assay type** | **Forward primer** | **Reverse primer** |
| --- | --- | --- | --- | --- | --- | --- |
| ACE | I/D | rs4340 | D > I | PCR, AGE | 5’-GAGCCACTCCCATCCTTTCT-3’ | 5’-GGCCATCACATTCGTCAGAT-3’ |
| AMPD1 | Q12X | rs17602729 | C > T | RFLP - HpyCH4IV | 5’-ATAGCCATGTTTCTGAATTA-3’ | 5’-GCAATCTACATGTGTCTACC-3’ |
| GDF8 | K153R | rs1805086 | A > G | RFLP - PspOMI | 5’-CATGTCAAGTTTCAGAGATCGG-3’ | 5’-CTAATGCAAGTGGATGGAAAACCC-3’ |
| HFE | H63D | rs1799945 | C > G | RFLP - BclI | 5’-GCCTGTTGCTCTGTCTCCAGG-3’ | 5’-CTCAGCTGCAGCCACATCTGG-3’ |
| PPARGC1A | G482S | rs8192678 | G > A | RFLP - MspI | 5’-TGTCATCAAACAGGCCATCCATGG-3’ | 5’-CCGAGCTGAACAAGCACTTCGG-3’ |
| ACTN3 | R577X | rs1815739 | C > T | HRM | 5’-GCACGATCAGTTCAAGGCAACAC-3’ | 5’-CTGAGGGTGATGTAGGGATTGGTG-3’ |
| CKMM | NcoI RFLP | rs8111989 | A > G | HRM | 5’-TGCATTTCAGTGTGGCCTTGAG-3’ | 5’-TCATCCCTCTGTAGCTGCTGCC-3’ |

ACE – Amplicons of 182bp for the D allele and 470bp for the I allele were obtained using the above primers (slightly modified from Lea et al., 2005). All fragments were separated by electrophoresis at 70V for 60 min on 2% agarose gels, 0.005% Ethidium Bromide. Positive controls for each genotype were sequenced by the Australian Genome Research Facility Ltd (AGRF), Brisbane, Australia.

AMPD1 – A PCR amplicon of 197bp was obtained using the above primers (obtained from Tsujino et al 1995[TSUJ1995]). PCR products digested with *Hpy*CH4IV restriction enzyme (New England Biolabs Inc., Ipswich, MA, USA), yielding C allele digestion into 110bp and 87bp fragments while T allele remained undigested. Restriction fragments were separated by electrophoresis at 75V for 90 min on 3.5% agarose gels, 0.005% Ethidium Bromide. Positive controls for CC and CT genotypes were sequenced by the Australian Genome Research Facility Ltd (AGRF), Brisbane, Australia; positive controls for the TT genotype were not obtained due to low genotype population frequency (HapMap CEU – T allele frequency 0.13, expected TT genotype frequency 0.017). Two TT individuals were observed in the cohort; these were confirmed by sequencing.

GDF8 – A PCR amplicon of 207bp was obtained using the above primers, which were manually designed for this study and checked for specificity and melting characteristics using the UCSC *in silico* PCR tool (Karolch*ik et a*l., 2008), NCBI BLAST (Altsch*ul et a*l., 1990), and DINAmelt (Markham & Zuker, 2005). PCR products digested with *Psp*OMI (New England Biolabs Inc., Ipswich, MA, USA), yeilding G allele digestion into 127bp and 80bp fragments, while the A allele remained undigested. Restriction fragments were separated by electrophoresis at 90V for 90 min on 2% agarose gels, 0.005% Ethidium Bromide. Positive controls for AA and AG genotypes were sequenced by the Australian Genome Research Facility Ltd (AGRF), Brisbane, Australia; positive controls for the GG genotype were not obtained due to low genotype population frequency (HapMap CEU – G allele frequency 0.017, expected GG genotype frequency 0.0003). No GG individuals were observed in the cohort; only nine AG individuals were observed. To ensure complete digestion of products despite the lack of GG individuals, an alternate amplicon containing a non-polymorphic *Psp*OMI restriction site (forward 5’-TGAAGGAGAAGGTGTCTGCGG-3’; reverse 5’-AGGACGGTGCGGTGAGAGTG-3’) was included in every digestion as a positive control; this amplicon completely digested into fragments of 146bp and 52bp in every run.

HFE – A PCR amplicon of 207bp was obtained using the above primers, which were manually designed for this study and checked for specificity and melting characteristics using the UCSC *in silico* PCR tool (Karolch*ik et a*l., 2008), NCBI BLAST (Altsch*ul et a*l., 1990), and DINAmelt (Markham & Zuker, 2005). PCR products were digested with *Bcl*I (New England Biolabs Inc., Ipswich, MA, USA), yielding C allele digestion into 127bp and 80bp fragments, while the G allele remained undigested. Restriction fragments were separated by electrophoresis at 75V for 90 min on 3.5% agarose gels, 0.005% Ethidium Bromide. Positive controls for CC and CG genotypes were sequenced by the Australian Genome Research Facility Ltd (AGRF), Brisbane, Australia; positive controls for the GG genotype were not obtained due to low genotype population frequency (HapMap CEU – G allele frequency 0.179, expected GG genotype frequency 0.032). Two GG individuals were observed in the cohort; these were confirmed by sequencing.

PPARGC1A – A PCR amplicon of 162bp was obtained using the above primers, which were manually designed for this study and checked for specificity and melting characteristics using the UCSC *in silico* PCR tool (Karolch*ik et a*l., 2008), NCBI BLAST (Altsch*ul et a*l., 1990), and DINAmelt (Markham & Zuker, 2005). PCR products were digested with *Msp*I (New England Biolabs Inc., Ipswich, MA, USA), yielding G allele digestion into 96bp and 66bp fragments, while the A allele remained undigested. Restriction fragments were separated by electrophoresis at 75V for 90 min on 3.5% agarose gels, 0.005% Ethidium Bromide. Positive controls for each genotype were sequenced by the Australian Genome Research Facility Ltd (AGRF), Brisbane, Australia.

ACTN3 – A PCR amplicon of 138bp was obtained using the above primers, designed by us for a previous study(Grea*ly et a*l., 2013). PCR products were amplified in the presence of a fluorescent dye (SYTO9, Invitrogen), which incorporates into DNA and allows fluorescence-based detection of amplicon melt temperatures during High Resolution Melt (HRM) analysis. PCR amplification and HRM analysis was conducted in a closed-tube reaction on a Rotorgene 6000 (Qiagen) with each sample typed in duplicate. Genotypes were determined by differential melt curves which clustered around positive controls, using the Rotor-Gene™ 6000 Series Software 1.7. Positive controls were validated using the previously reported *ACTN3* R577X RFLP assay(North & Beggs, 1996) involving restriction enzyme digestion with *Dde*I (New England Biolabs Inc., Ipswich, MA, USA). The 138bp amplicon contained a non-polymorphic *Dde*I restriction site which resulted in a 4bp band regardless of allele type (not visible on AGE). T allele digestion yielded two further fragments of 97bp and 37bp, while the C allele was undigested, resulting in a 134bp fragment. Restriction fragments were separated by electrophoresis at 75V for 90 min on 3% agarose gels, 0.005% Ethidium Bromide. Positive control genotypes determined by HRM were 100% concordant with genotypes determined using RFLP.

CKMM – a PCR amplicon of 115bp was obtained using the above primers, which were manually designed for this study and checked for specificity and melting characteristics using the UCSC *in silico* PCR tool (Karolch*ik et a*l., 2008), NCBI BLAST (Altsch*ul et a*l., 1990), and DINAmelt (Markham & Zuker, 2005). PCR products were amplified in the presence of a fluorescent dye (SYTO9, Invitrogen), which incorporates into DNA and allows fluorescence-based detection of amplicon melt temperatures during HRM analysis. PCR amplification and HRM analysis was conducted in a closed-tube reaction on a Rotorgene 6000 (Qiagen) with each sample typed in duplicate. Genotypes were determined by differential melt curves which clustered around positive controls, using the Rotor-Gene™ 6000 Series Software 1.7. Positive controls were validated using an RFLP assay involving restriction enzyme digestion with *NcoI*HF (New England Biolabs Inc., Ipswich, MA, USA). A allele digestion yielded fragments of 63bp and 52bp, while the G allele was undigested, resulting in a 115bp fragment. Restriction fragments were separated by electrophoresis at 75V for 90 min on 3.5% agarose gels, 0.005% Ethidium Bromide. Positive control genotypes determined by HRM were 100% concordant with genotypes determined using RFLP.
