# Supplementary material for: Evaluation of a 7-Gene Genetic Profile for Athletic Endurance Phenotype in Ironman Championship Triathletes
Source: PLoS One. 2015 Dec 30;10(12):e0145171. doi: 10.1371/journal.pone.0145171 (PMC4696732; doi:10.1371/journal.pone.0145171)
Supplement: S2 Table — (DOC) [file pone.0145171.s003.doc]

**S2 Table: χ^2^ testing for conformation to Hardy-Weinberg Equilibrium (HWE)**

| **Marker** | **HWE (HapMap)** | **HWE (all)** | **HWE (top)** | **HWE (bottom)** |
| --- | --- | --- | --- | --- |
| ACE | χ^2^ = 0.0076  p = 0.9305 | χ^2^ = 0.3650  p = 0.5457 | χ^2^ = 0.0923  p = 0.7613 | χ^2^ = 2.9514  p =0.0858 |
| ACTN3 | χ^2^ = 3.2261  p = 0.0725 | χ^2^ = 0.0002  p = 0.9895 | χ^2^ = 0.5294  p = 0.4668 | χ^2^ = 0.0525  p = 0.8188 |
| AMPD1 | χ^2^ = 0.6796  p = 0.40972 | χ^2^ = 0.4005  p = 0.5268 | χ^2^ = 0.0664  p = 0.7966 | χ^2^ = 0.6168  p = 0.4322 |
| CKMM | χ^2^ = 1.1356  p = 0.2866 | χ^2^ = 0.0544  p = 0.8157 | χ^2^ = 1.6095  p = 0.2046 | χ^2^ = 3.1922  p = 0.0740 |
| GDF8 | χ^2^ = 0.0172  p = 0.8955 | χ^2^ = 0.1088  p = 0.7415 | χ^2^ = 0.0000  p = 1.0000 | χ^2^ = 0.0156  p = 0.9006 |
| HFE | χ^2^ = 2.6465  p = 0.1038 | χ^2^ = 1.3233  p = 0.2500 | χ^2^ = 0.3022  p = 0.5825 | χ^2^ = 0.3265  p = 0.5677 |
| PPARGC1A | χ^2^ = 1.7447  p = 0.1865 | χ^2^ = 2.2028  p = 0.1378 | χ^2^ = 0.0598  p = 0.8069 | χ^2^ = 1.2524  p = 0.2631 |

Observed genotype frequencies for each group (as per Table 1 in the main text) were used to calculate both the observed allele frequencies (p and q) and the expected genotype frequencies according to the HWE formula p^2^ + 2pq + q^2^. Observed genotype frequencies and expected Hardy-Weinberg genotype frequencies were compared by calculation of a χ^2^ statistic, and p-values were obtained assuming 1 d.f. for each test. Confidence level α = 0.05; all p-values were greater than α indicate that the observed genotype frequencies are not significantly different from expected frequencies under HWE.
