# Supplementary material for: Evaluation of a 7-Gene Genetic Profile for Athletic Endurance Phenotype in Ironman Championship Triathletes
Source: PLoS One. 2015 Dec 30;10(12):e0145171. doi: 10.1371/journal.pone.0145171 (PMC4696732; doi:10.1371/journal.pone.0145171)
Supplement: S3 Table — (DOC) [file pone.0145171.s004.doc]

**S3 Table: χ^2^ testing of genotype frequencies within age, sex, and ethnicity groups**

| **Marker** | **Sex (M v F)** | **Continent** | **Caucasian** | **Age (ANOVA) F, p** |
| --- | --- | --- | --- | --- |
| ACE | χ^2^ = 0.2217  p = 0.8951 | Fisher’s exact  p = 0.844 | Fisher’s exact  p = 0.538 | F = 0.360  p = 0.698 |
| ACTN3 | χ^2^ = 3.579  p = 0.1670 | Fisher’s exact  p = 0.633 | Fisher’s exact  p = 0.745 | F = 0.167  p = 0.846 |
| AMPD1 | Fisher’s exact  p = 0.7001 | Fisher’s exact  p = 0.937 | Fisher’s exact  p = 1.000 | F = 1.657  p = 0.193 |
| CKMM | χ^2^ = 5.8926  p = 0.0525 | Fisher’s exact  p = 0.559 | Fisher’s exact  p = 0.530 | F = 1.646  p = 0.195 |
| GDF8 | Fisher’s exact  p = 0.7060 | Fisher’s exact  p = 0.420 | Fisher’s exact  p = 0.352 | F = 0.097  p = 0.755 |
| HFE | Fisher’s exact  p = 0.2569 | Fisher’s exact  p = 0.897 | Fisher’s exact  p = 0.499 | F = 0.182  p = 0.834 |
| PPARGC1A | χ^2^ = 3.3925  p = 0.1834 | Fisher’s exact  p = 0.597 | Fisher’s exact  p = 0.911 | F = 0.318  p = 0.728 |

Genotype frequencies were determined for each category of sex (male, female); continent of origin (North America, Europe, Oceania, Africa, Asia, South America); and Caucasian (North America/Europe/Oceania, Africa/Asia/South America). Genotype frequencies were compared between groups for each variable using a χ^2^ test and obtaining p-values for the approproiate d.f.; however, when expected genotype frequencies were found to be less than 5 in more than 20% of cells, Fisher’s Exact test was employed instead (as indicated in Table 2 above). Confidence level α = 0.05; all p-values were greater than α indicating that the observed genotype frequencies are not significantly different among either males and females, among athletes of differing continents, and among athletes of differing age groups. This may be in part due to small sample sizes of some of the groups, such as age group 70+ (n=5) and continent of origin Africa (n=1), Asia (n=2), and South America (n=6). In order to improve power and increase sample size per group, the variable ‘continent of origin’ was collapsed into a two-group variable ‘Caucasian’, denoting predominantly Caucasian athletes from non-Caucasian athletes. No significant difference in genotype frequency was detected between these groups either. Additionally, the variable ‘age’ was also analysed as a continuous variable by one-way analysis of variance (ANOVA); no significant difference in mean age among genotype groups was detected for any marker. These results indicate that genotype distribution is not significantly different among these groups and that data may be analysed in total to improve power of the main analyses. For genotype distribution among male and female athletes, see S4 Table; for age distribution among genotype groups, see S5 Table.
