# Supplementary material for: Evaluation of a 7-Gene Genetic Profile for Athletic Endurance Phenotype in Ironman Championship Triathletes
Source: PLoS One. 2015 Dec 30;10(12):e0145171. doi: 10.1371/journal.pone.0145171 (PMC4696732; doi:10.1371/journal.pone.0145171)
Supplement: S4 Table — (DOC) [file pone.0145171.s005.doc]

**S4 Table: Genotype distribution within male and female cohort athletes**

| **Gene** | **rsID** | **Genotype** |  | **Genotype frequency, n (%)** | | **χ^2^ p** |
| --- | --- | --- | --- | --- | --- | --- |
|  |  |  |  | **Male athletes** | **Female athletes** |  |
| ACE | rs4340 | D/D |  | 62 (43.4%) | 21 (39.6%) | 0.8951 |
|  |  | I/D |  | 66 (46.2%) | 26 (49.1%) |  |
|  |  | I/I |  | 15 (10.5%) | 6 (11.3%) |  |
| ACTN3 | rs1815739 | R/R |  | 43 (30.1%) | 9 (17.0%) | 0.1670 |
|  |  | R/X |  | 67 (46.9%) | 31 (58.5%) |  |
|  |  | X/X |  | 33 (23.1%) | 13 (24.5%) |  |
| AMPD1 | rs17602729 | Q/Q |  | 110 (76.9%) | 39 (75.0%) | exact 0.7001 |
|  |  | Q/X |  | 32 (22.4%) | 12 (23.1%) |  |
|  |  | X/X |  | 1 (0.7%) | 1 (1.9%) |  |
| CKMM | rs8111989 | A/A |  | 62 (43.4%) | 31 (58.5%) | 0.0525 |
|  |  | A/G |  | 68 (47.6%) | 15 (28.3%) |  |
|  |  | G/G |  | 13 (9.1%) | 7 (13.2%) |  |
| GDF8 | rs1805086 | K/K |  | 136 (95.8%) | 50 (94.3%) | exact 0.7060 |
|  |  | K/R |  | 6 (4.2%) | 3 (5.7%) |  |
|  |  | R/R |  | 0 (0.0%) | 0 (0.0%) |  |
| HFE | rs1799945 | H/H |  | 104 (74.8%) | 34 (65.4%) | exact 0.2569 |
|  |  | H/D |  | 33 (23.7%) | 18 (34.6%) |  |
|  |  | D/D |  | 2 (1.4%) | 0 (0.0%) |  |
| PPARGC1A | rs8192678 | G/G |  | 59 (41.5%) | 15 (28.3%) | 0.1834 |
|  |  | G/S |  | 56 (39.4%) | 28 (52.8%) |  |
|  |  | S/S |  | 27 (19.0%) | 10 (18.9%) |  |

Genotype frequencies were determined for each category of sex (male, female) and were compared between groups for each variable using a χ^2^ test or Fisher’s Exact test if expected cell counts were less than 5 in 20% or more cells. Confidence level α = 0.05; all p-values were greater than α indicating that the observed genotype frequencies are not significantly different among either males and females.
