# Supplementary material for: Evaluation of a 7-Gene Genetic Profile for Athletic Endurance Phenotype in Ironman Championship Triathletes
Source: PLoS One. 2015 Dec 30;10(12):e0145171. doi: 10.1371/journal.pone.0145171 (PMC4696732; doi:10.1371/journal.pone.0145171)
Supplement: S5 Table — (DOC) [file pone.0145171.s006.doc]

**S5 Table: Age distribution within genotype groups**

| **Gene** | **rsID** | **Genotype** | **n** | **Age in yrs - Mean (SE)** | **F, p** | **Levene p** |
| --- | --- | --- | --- | --- | --- | --- |
| ACE | rs4340 | D/D | 83 | 41.76 (1.34) | 0.360, 0.698 | 0.259 |
|  |  | I/D | 92 | 43.23 (1.10) |
|  |  | I/I | 21 | 42.57 (2.59) |
| ACTN3 | rs1815739 | R/R | 52 | 43.27 (1.66) | 0.167 , 0.846 | 0.729 |
|  |  | R/X | 98 | 42.41 (1.12) |
|  |  | X/X | 46 | 41.98 (1.72) |
| AMPD1 | rs17602729 | Q/Q | 149 | 42.95 (0.90) | 1.657, 0.193 | 0.823 |
|  |  | Q/X | 44 | 42.07 (1.92) |
|  |  | X/X | 2 | 28.50 (7.50) |
| CKMM | rs8111989 | A/A | 93 | 41.02 (1.17) | 1.646, 0.195 | 0.434 |
|  |  | A/G | 83 | 43.69 (1.30) |
|  |  | G/G | 20 | 44.80 (2.14) |
| GDF8 | rs1805086 | K/K | 186 | 42.55 (0.85) | 0.097, 0.755 | 0.050 |
|  |  | K/R | 9 | 41.33 (1.94) |
|  |  | R/R | 0 | n/a |
| HFE | rs1799945 | H/H | 138 | 42.14 (0.99) | 0.182 , 0.834 | 0.232 |
|  |  | H/D | 51 | 43.00 (1.50) |
|  |  | D/D | 2 | 45.50 (2.50) |
| PPARGC1A | rs8192678 | G/G | 74 | 43.31 (1.34) | 0.318, 0.728 | 0.956 |
|  |  | G/S | 84 | 41.87 (1.23) |
|  |  | S/S | 37 | 42.30 (1.95) |

Mean age in years and standard error was calculated for each genotype group for each marker and one-way analysis of variance (ANOVA) was performed to evaluate whether mean age was significantly different between any of the genotype groups. Confidence level α = 0.05; all p-values were greater than α indicating that there is no significant difference in mean age among genotype groups for any marker.
